# Implementing Lp(a) Screening in Veterans Affairs and Safety Net Settings: A Dual Site Pilot Quality Improvement Initiative

**DOI:** 10.1016/j.ajpc.2026.101777

**Published:** 2026-07-31

**Authors:** Erfan Tasdighi, Navid Radfar, Joseph Santitoro, Eugene Kim, Emaad Siddiqui, Meet Shah, Delia Cotiga, Daniel Matassa, Jared Walsh, Roger S. Blumenthal, Michael J. Blaha, Alfonso H. Waller

**Affiliations:** a Department of Internal Medicine, Rutgers New Jersey Medical School, Newark, NJ, USA; b Division of Cardiology, Department of Medicine, Rutgers New Jersey Medical School, Newark, New Jersey, USA; c Johns Hopkins Ciccarone Center for Prevention of Cardiovascular Disease, Baltimore, Maryland, USA

**Keywords:** Lipoprotein(a), Cardiovascular disease prevention, Health equity, Quality improvement, Universal screening, Safety net, Veterans Affairs, Dyslipidemia

## 1 Introduction

Lipoprotein(a) [Lp(a)] is an independent, causal, and largely genetically determined risk factor for atherosclerotic cardiovascular disease (ASCVD), and roughly one in five adults carries an elevated concentration.^1^ The 2019 National Lipid Association scientific statement, and subsequently the 2026 ACC/AHA/Multisociety Class 1 recommendation, endorsed one time Lp(a) measurement in all adults.^2^^,^^3^ Yet testing remains below 1% in Veterans Affairs (VA) populations^4^ and lowest in urban safety net settings serving racial and ethnic minorities.^5^ We describe two pragmatic quality improvement (QI) initiatives designed to increase Lp(a) testing and document early management after elevated results in these under screened settings.

## 2 Methods

We conducted two parallel QI initiatives in New Jersey, PATRIOT QI, in VA primary care at the East Orange VA (April 2024 to January 2025), a prospective clinician facing educational intervention. ACHILLES QI was a prospective single site initiative at University Hospital, Newark, an urban safety net academic center serving a predominantly Black and Hispanic population (June to December 2025). Eligible patients were adults 18 years and older seen at primary care visits (Fig. 1A).

**Fig. 1:**
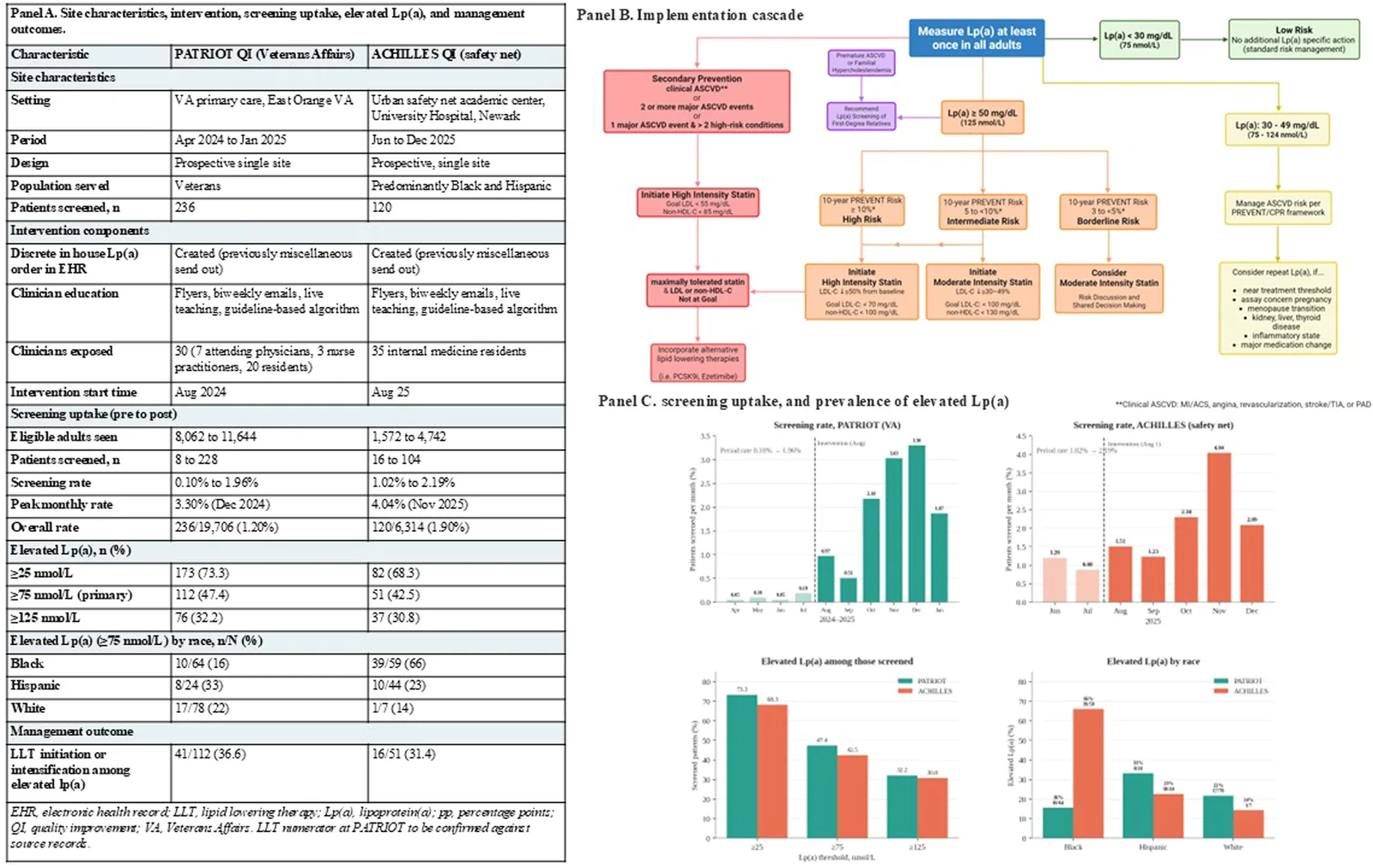
Panel A. Site characteristics, intervention, screening uptake, elevated Lp(a), and management outcomes. All values are expressed as number and percentage. **Panel B.** Implementation cascade and Lipoprotein(a)-guided lipid-lowering treatment pathway: a dual site quality improvement initiative pilot. Algorithm for stratifying Lp(a) concentration into low risk <30 mg/dL (<75 nmol/L), intermediate range 30-49 mg/dL (75-124 nmol/L), and elevated Lp(a) ≥ 50 mg/dL (≥125 nmol/L) and integrating results with clinical ASCVD status and 10-year PREVENT-ASCVD risk to guide lipid-lowering therapy intensity, LDL-C/non-HDL-C treatment goals, and consideration of first-degree relative screening. PREVENT-ASCVD risk categories (marked with asterisks) and lipid treatment thresholds are based on the 2026 ACC/AHA Guideline on the Management of Dyslipidemia. Created in BioRender. Kim, E. (2026) https://BioRender.com/99o8vqt. **Panel C.** Lp(a) screening uptake, prevalence of elevated Lp(a) in total population and across race in Veterans Affairs and safety net settings: Monthly Lp(a) screening rate (patients screened per month) at each site, with preintervention months shaded lighter and the dashed line marking the intervention start. Elevated Lp(a) is defined as Lp(a) ≥ 75 nmol/L. Abbreviations: EHR, electronic health record; LLT, lipid lowering therapy; Lp(a), lipoprotein(a); pp, percentage points; QI, quality improvement; VA, Veterans Affairs; ACS, acute coronary syndrome; ASCVD, atherosclerotic cardiovascular disease; CPR, cardiovascular prevention risk framework; HDL-C, high-density lipoprotein cholesterol; LDL, low-density lipoprotein; LDL-C, low-density lipoprotein cholesterol; MI, myocardial infarction; non-HDL-C, non–high-density lipoprotein cholesterol; PAD, peripheral artery disease; PCSK9i, proprotein convertase subtilisin/kexin type 9 inhibitor; PREVENT, Predicting Risk of Cardiovascular Disease EVENTs; TIA, transient ischemic attack.

The intervention shared a common core across sites but differed in intensity. At both sites, the principal intervention made Lp(a) a distinct, searchable in-house laboratory order within the electronic health record (HER); at baseline it could be requested only as a miscellaneous send out, a substantial barrier to routine ordering. Both sites also delivered a clinician facing educational package comprising printed flyers, biweekly emails, live teaching sessions, and a guideline based Lp(a) screening algorithm that specified the recommended response to an elevated result. The sites differed in reach: at ACHILLES the intervention was directed solely at 35 internal medicine residents whereas at PATRIOT it engaged a broader primary care ordering base spanning 30 clinicians including 7 attending physicians, 3 nurse practitioners, and 20 residents, producing a more varied group of exposed clinicians. The implementation cascade has been demonstrated in Fig. 1B.

The primary outcome was the change in Lp(a) testing rate before versus after the intervention. Secondary outcomes were the prevalence of elevated Lp(a) among those screened and, among patients with an elevated result, initiation or intensification of lipid lowering therapy (LLT) within 6 months, ascertained from the EHR; lifestyle counseling, routine at internal medicine visits, was not counted as Lp(a) directed management. Elevated Lp(a) was prespecified as 75 nmol/L or greater, with 25 and 125 nmol/L thresholds reported secondarily. Both initiatives were reviewed as QI activities by the oversight bodies at the East Orange VA and Rutgers New Jersey Medical School.

## 3 Results

**Screening uptake.** At PATRIOT, during the preintervention period (April to July 2024), 8,062 primary care patients were seen, of whom 8 were screened by 5 clinicians (0.10%); after the intervention began in August 2024 (August 2024 to January 2025), 11,644 were seen, of whom 228 were screened by all 30 clinicians (1.96%), a 19.7 fold rise in the screening rate (absolute increase 1.86 percentage points) that peaked at 3.30% in December 2024. Across the full period, 236 of 19,706 patients were screened (1.20%).

At ACHILLES, in total, 120 Lp(a) tests were ordered over 7 months. During the preintervention period (June to July 2025), 1,572 eligible adults were seen, of whom 16 were screened (1.02%); after the intervention began on 1 August 2025 (August to December 2025), 4,742 eligible adults were seen, of whom 104 were screened (2.19%), an approximately twofold rise in the screening rate that peaked at 4.04% (34 of 842) in November (Fig. 1C).

**Elevated Lp(a) across race and ethnicity and management.** At the prespecified primary threshold of 75 nmol/L or greater, elevated Lp(a) was present in 112 of 236 patients (47.4%) at PATRIOT and 51 of 120 (42.5%) at ACHILLES. At the secondary thresholds of 25 and 125 nmol/L or greater, prevalence was 73.3% (173) and 32.2% (76) at PATRIOT and 68.3% (82) and 30.8% (37) at ACHILLES, respectively.

At PATRIOT, elevated Lp(a) was most common among Hispanic patients: 8 of 24 Hispanic patients tested (33%) had an elevated level, compared with 17 of 78 White patients (22%) and 10 of 64 Black patients (16%). At ACHILLES, elevated Lp(a) was most common among Black patients: 39 of 59 Black patients tested (66%) had an elevated level, compared with 10 of 44 Hispanic patients (23%) and 1 of 7 White patients (14%), consistent with the higher average Lp(a) concentrations reported in individuals of African ancestry.^6^ Among patients with elevated Lp(a), LLT was initiated or intensified in 41 of 112 (36.6%) at PATRIOT and 16 of 51 (31.4%) at ACHILLES (Fig. 1C)**.**

## 4 Discussion

In two under screened settings, a low cost, clinician facing intervention paired with a single structural change, establishing Lp(a) as a discrete in-house order, increased testing in both a VA system and an urban safety net center. Elevated Lp(a) was common among those tested, though this reflects a clinician selected, likely risk enriched sample rather than the prevalence among all adults in either system.

These results extend a growing literature establishing Lp(a) as a clinically actionable biomarker and complement the 2026 Dyslipidemia Guideline by addressing the implementation gap it creates. While elevated Lp(a) is not yet modifiable by an approved Lp(a) specific therapy, detection enables intensified management of global ASCVD risk, including more aggressive LDL-cholesterol lowering, identification of individuals who warrant earlier or more intensive preventive treatment, and cascade screening of relatives. A practical, scalable screening pathway in high-risk populations is a prerequisite for equitable access to any future Lp(a) directed treatment.

Because the intervention relied on clinician education, a guideline-based algorithm, and integration of Lp(a) ordering into the electronic medical record at minimal marginal cost, the framework should be portable across payer structures, staffing models, and patient populations, offering a replicable template for closing the Lp(a) implementation gap.

Key questions remain. Downstream management was incomplete, particularly at the safety net site, where fewer than one third of patients with an elevated result had lipid lowering therapy initiated or intensified. Because ordering was discretionary, we cannot confirm that every eligible adult was offered screening, so the initiatives increased guideline concordant testing rather than achieving universal reach. Although elevated Lp(a) is not yet modifiable by an approved Lp(a) specific therapy, detection enables intensified management of global ASCVD risk, including more aggressive LDL cholesterol lowering and cascade screening of relatives, and positions patients for future Lp(a) directed therapies. Structured management and cascade screening pathways are therefore needed to convert detection into consistent preventive care.

## 5 Limitations

This study has several limitations. The Lp(a) order was at the discretion of the clinicians; therefore, the tested sample was likely risk enriched, and the high prevalence of elevated Lp(a) reflects those tested rather than all adults in these systems. Follow up was short, order completion was not captured, and each initiative was single site, limiting generalizability. Without prior testing rates by race, race stratified denominators, and longitudinal follow up, we cannot determine whether screening disparities were truly reduced.

## 6 Conclusions

In two New Jersey VA and safety net settings, low-cost clinician education and EMR workflow support were associated with increased Lp(a) testing and documented management in some patients with elevated results. These findings support further evaluation of scalable QI strategies for universal Lp(a) screening, standardized management pathways, and longer term follow up.

## Declarations

### Disclosures

Michael J. Blaha disclosed Novartis, Novo Nordisk, Eli Lilly, Merck, Boehringer Ingelheim, New Amsterdam, and Idorsia. Other authors report no disclosures.

### Ethics

Both initiatives were conducted as QI activities and reviewed by the responsible oversight bodies at the East Orange VA and Rutgers New Jersey Medical School.

## Data availability

Data cannot be shared under Veterans Affairs healthcare policies.

## Abbreviations

ASCVD, atherosclerotic cardiovascular disease; EHR, electronic health record; EMR, electronic medical record; Lp(a), lipoprotein(a); LLT, lipid lowering therapy; QI, quality improvement; VA, Veterans Affairs.

## Author Agreement Statement

We the undersigned declare that this manuscript is original, has not been published before and is not currently being considered for publication elsewhere.

We confirm that the manuscript has been read and approved by all named authors and that there are no other persons who satisfied the criteria for authorship but are not listed. We further confirm that the order of authors listed in the manuscript has been approved by all of us.

We understand that the Corresponding Author is the sole contact for the Editorial process. He/she is responsible for communicating with the other authors about progress, submissions of revisions and final approval of proofs

## CRediT authorship contribution statement

**Erfan Tasdighi:** Writing – review & editing, Writing – original draft, Formal analysis, Data curation, Conceptualization. **Navid Radfar:** Writing – review & editing, Writing – original draft, Conceptualization. **Joseph Santitoro:** Writing – original draft, Data curation. **Eugene Kim:** Visualization, Data curation. **Emaad Siddiqui:** Writing – review & editing, Conceptualization. **Meet Shah:** Writing – review & editing, Conceptualization. **Delia Cotiga:** Writing – review & editing, Supervision, Conceptualization. **Daniel Matassa:** Writing – review & editing, Supervision, Data curation, Conceptualization. **Jared Walsh:** Writing – review & editing, Supervision, Data curation, Conceptualization. **Roger S. Blumenthal:** Writing – review & editing, Validation, Conceptualization. **Michael J. Blaha:** Writing – review & editing, Validation, Conceptualization. **Alfonso H. Waller:** Writing – review & editing, Validation, Conceptualization.

## Declaration of competing interest

The authors declare that they have no known competing financial interests or personal relationships that could have appeared to influence the work reported in this paper.
